# The Immunosuppressive Effect of TNFR2 Expression in the Colorectal Cancer Microenvironment

**DOI:** 10.3390/biomedicines11010173

**Published:** 2023-01-10

**Authors:** Nurul Hakimah Mohd Salim, Ali Mussa, Naveed Ahmed, Suhana Ahmad, Chan Yean Yean, Rosline Hassan, Vuk Uskoković, Rohimah Mohamud, Nur Asyilla Che Jalil

**Affiliations:** 1 Department of Pathology, School of Medical Sciences, Universiti Sains Malaysia, Kubang Kerian 16150, Kelantan, Malaysia; 2 Department of Haematology, School of Medical Sciences, Universiti Sains Malaysia, Kubang Kerian 16150, Kelantan, Malaysia; 3 Department of Biology, Faculty of Education, Omdurman Islamic University, Omdurman P.O. Box 382, Sudan; 4 Department of Medical Microbiology and Parasitology, School of Medical Sciences, Universiti Sains Malaysia, Kubang Kerian 16150, Kelantan, Malaysia; 5 Department of Immunology, School of Medical Sciences, Universiti Sains Malaysia, Kubang Kerian 16150, Kelantan, Malaysia; 6 TardigradeNano LLC, Irvine, CA 92604, USA; 7 Department of Mechanical Engineering, San Diego State University, San Diego, CA 92182, USA

**Keywords:** colorectal cancer, TNFR2, Treg cells, MDSC, TNF, immunosuppressive, tumor microenvironment

## Abstract

Colorectal cancer (CRC) represents one of the most common causes of death among cancers worldwide. Its incidence has been increasing among the young population. Many risk factors contribute to the development and progression of CRC and about 70% of them are sporadic. The CRC microenvironment is highly heterogeneous and represents a very complex immunosuppressive platform. Many cytokines and their receptors are vital participants in this immunosuppressive microenvironment. Tumor necrosis factors (TNFs) and TNF receptor 2 (TNFR2) are critical players in the development of CRC. TNFR2 was observed to have increased the immunosuppressive activity of CRC cells via regulatory T cells (T regs) and myeloid-derived suppressor cells (MDSC) in the CRC microenvironment. However, the exact mechanism of TNFR2 in regulating the CRC prognosis remains elusive. Here, we discuss the role of TNFR2 in immune escape mechanism of CRC in the immunosuppressive cells, including Tregs and MDSCs, and the complex signaling pathways that facilitate the development of CRC. It is suggested that extensive studies on TNFR2 downstream signaling must be done, since TNFR2 has a high potential to be developed into a therapeutic agent and cancer biomarker in the future.

## 1. Introduction: Overview of Colorectal Cancer

Colorectal cancer (CRC) is the third most common cancer in men after lung and prostate, with about 1.06 million new cases reported in 2020. It is the second most common cancer in women after breast cancer, with a reported 865,630 new cases [1]. In the past, CRC has primarily affected the elderly and was often diagnosed in the late stages of the disease; its prevalence among young people is currently growing [2]. CRC is a heterogeneous disease and about 10% of all adenomas develop into invasive cancer; 95% of all CRC that results from these adenomatous polyps is referred to as an adenocarcinoma [3]. CRC develops over time, approximately 10 to 15 years, due to a series of well-defined signaling pathways and genetic and epigenetic changes that lead to the inactivation and activation of tumor suppressor genes and oncogenes, respectively. Additionally, CRC is caused by three key mechanisms: chromosomal instability (CIN), microsatellite instability (MSI), and the CpG island methylator phenotype (CIMP). Cancer genome aberrations and the formation of an inflammatory microenvironment are critical in the development and progression of CRC [4,5]. Moreover, a favorable family history is seen in 10–20% of CRC patients. A detailed family history is critical in attaining a diagnosis, since it provides the most effective monitoring tool for CRC prevention. Non-polyposis (Lynch) and polyposis (Familial adenomatous polyposis) syndromes are the two types of hereditary CRC. Furthermore, modifiable variables and environmental lifestyles such as smoking, obesity, sedentary lifestyle, alcohol use, and a high diet of processed meat may raise the risk of CRC. The signs and symptoms vary among patients, but the most common symptoms are rectal bleeding, changes in bowel habit, anemia, or abdominal pain. The current screening options such as fecal occult blood test (FOBT), endoscopy, colonoscopy and CT colonoscopy are available for patients, depending on the patient’s needs and condition. Surgery is still the “gold standard” for CRC treatment and post-operative chemotherapy, immunotherapy, and radiotherapy may help to further improve patient survival and prevent cancer relapse [6].

In a normal microenvironment, to maintain immunological homeostasis, the immune system is activated during pathogen invasion, which results in the removal of such invaders from the host as well as the prevention of tumor formation via apoptosis. This initial reaction to pathogens relies on innate immunity, which is essential in protecting the host before adaptive immune responses arise. Unlike innate immunity, adaptive immunity develops in response to infection and detects a wide range of microbial and non-microbial agents [7]. Humoral immunity and cell-mediated immunity are two types of adaptive immunity that are mediated by different types of lymphocytes and eradicate various microbes. Cell-mediated immunity is mediated by T lymphocytes and their products such as cytokines, CD4^+^ T lymphocytes that assist macrophage to consume microorganisms and C8^+^ CTL to make antibodies. But certain T lymphocytes such as regulatory T cells (Treg) have a function in suppressing immune response [7]. Meanwhile, antibodies generated by B lymphocytes, plasma cells, and defense mechanisms against external microorganisms drive the humoral response [7]. Immune cells also detect tumor-specific antigens or chemicals that are produced as a result of cellular stress.

**Figure 1:**
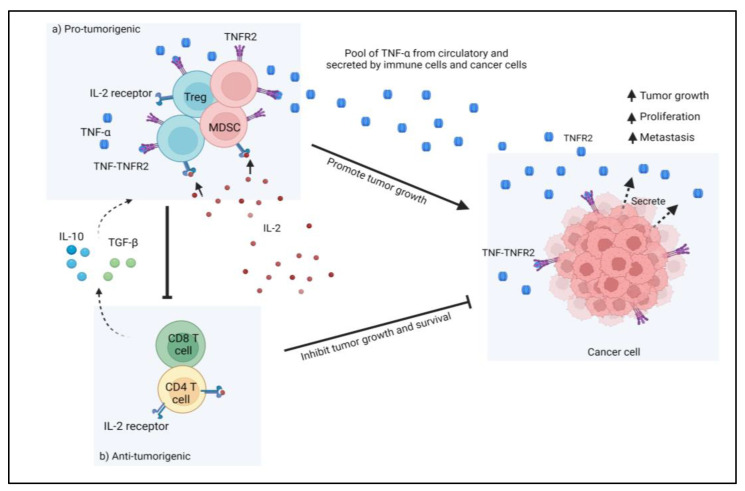
In tumor microenvironments (TME), immunosuppressive cells such as Treg cells and MDSCs suppress the function of anti-tumorigenic cells such as T cells and effector T cells. Treg cells compete with T cells for IL-2, which is necessary for T cell activation. Additionally, the presence of the cytokines IL-10 and TGF-β also increases the activation, proliferation, and suppressive activities of Tregs and MDSCs, hence increasing their IL-2 uptake. Furthermore, the presence of TNFR2 on Tregs, MDSCs, and tumor cells creates a favorable environment for tumor survival and growth via an immune escape mechanism.

In contrast, this stress is detrimental in the tumor microenvironment (TME), where it leads to immune escape mechanisms of tumor cells and promotes tumor development. Additionally, when immune cells such as regulatory T cells (Treg) and myeloid-derived suppressive cells (MDSC) are enriched within the TME, they will dampen the activity of anti-tumor effectors and encourage tumor development. Moreover, the presence of Tregs and MDSCs has a significant suppressive role by inhibiting effector T cell activities and reducing CD4^+^ T cells and effector CD8^+^ T cells anti-tumor activation. Apart from that, other factors such as cell-surface proteins, cytokine/chemokine, transcriptional factors, and enzymes may contribute to the immunosuppressive role in the TME of CRC. These factors have the potential to be used as immunotherapy targets, such as PD-1/PD-L1, CTLA-4, which is expressed on Treg. They produced promising outcomes in CRC. Besides, interleukin-2 (IL-2) is important in CRC TME, where it is essential for the activation and proliferation of conventional T cells (Tconv) as well as the maintenance of Treg function and phenotype. However, Tregs do not make IL-2 on their own as they require external IL-2 for cell survival by increasing the affinity of the IL-2 receptor (CD25) to capture IL-2 from the environment, and so the amount of IL-2 for conventional T cells will be reduced [8]. Cancer cells boost their potential for IL-2 absorption to evade the immune system; the presence of the cytokines IL-10 and TGF-β also promotes the activation, proliferation, and suppressive actions of Tregs and MDSCs, thereby enhancing their IL-2 uptake and creating conditions that are more conducive to tumor development and metastasis. Figure 1 shows the action of immunosuppressive cells in TME that led to cancer progression. Furthermore, CRC tumor cells also produce TNFR2 on their surface, which interacts with TNF-α and promotes tumor cell proliferation in TME. However, studies are still ongoing, as TME immunity is complicated. The complexities of TME immunity encouraged us to look into the role of TNFR2 in CRC immune resistance via its expression in various cell types and signaling pathways that promote CRC development.

## 2. Tumor Necrosis Factor (TNF) and Its Receptors (TNFR1/2): Biology and Signaling Pathway

TNF was coined as a term in the early 1970s [9]. It is primarily produced by monocytes/macrophages, and is a multifunctional cytokine that is involved in the control of inflammation, the development of secondary lymphoid organs, and immune regulation [10]. The functions of TNF were associated with the different engagement of its two receptors, TNFR1 and TNFR2, which are differentially expressed on various cell types including cancer cells. TNFR1 (p55) is expressed on almost all cell types, whereas TNFR2 (p75) expression is restricted to certain cell types, such as neurons, oligodendrocytes, MDSC, Tregs, and monocytes [11,12,13,14,15]. Additionally, TNFR1 may be activated upon binding to either membrane-bound or soluble TNFs (sTNF), whereas TNFR2 is primarily activated by transmembrane homotrimeric protein (tmTNF) that can be proteolytically processed into sTNF form by TNF-α-converting enzyme (TACE).

TNFR1 is a death domain (DD)-containing receptor that may promote the expression of proinflammatory genes while also inducing two forms of programmed cell death under certain conditions. In contrast, TNFR2 is associated with cell proliferation and survival [16] and has lately received a lot of interest due to its role in the maintenance of the Treg compartment and also in regard to cancers [17,18]. In contrast to TNFR1, TNFR2 does not have intracellular DD and it is activated by mTNF. TNFR2 interacts directly with TNFR-associated factors 1, 2, and 3 (TRAF 1, 2, and 3) to recruit cellular inhibitors of apoptosis 1 and 2 (cIAP1 and 2). This complex process promotes cell survival signaling through the formation of complex I, inducing the activation of NF-κB, MAPK, and AKT signaling, thus promoting the proliferation of cells and tissue renewal. TNF–TNFR2 binding also activates the P13K/AKT signaling pathway to maintain cell survival and enhance their proliferation. Even though TNFR2 does not have DD, the activation of caspase and cell apoptosis can be triggered by the interaction of the intercellular domain with signal complex II under specific stress conditions or when cIAP pools are depleted [19].

**Figure 2:**
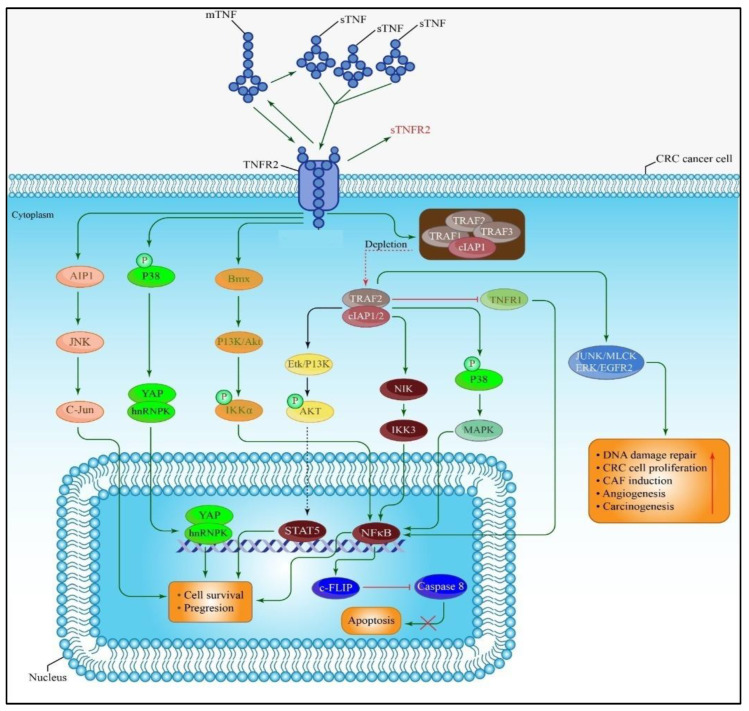
Signaling pathway of TNFR2 in TME. The binding of TNF to TNFR2 activates several signals associated with the growth, proliferation, and survival of the tumor.

TNFR2 activation is largely dependent on transmembrane TNF expressed on surrounding cells [20] and sTNF interaction with TNFR2 failed to trigger effective receptor signaling [21]. Furthermore, the binding of TNFR2 to bone marrow X-linked kinase (Bmx) promotes the Akt signaling pathway and controls the TNFR2-mediated NF-kB signaling pathway [22,23]. Furthermore, activation of NF-kB by TNFR2 leads to rapid tumor growth via mitogen-activated protein kinase (MAPK) signaling [24,25]. Surprisingly, the binding of membrane-bound TNF to TNFR2 can result in the phosphorylation of the STAT5 via P13/Akt signaling, which impairs Th17 differentiation [26]. Moreover, TNFR2 stimulates the p13/Akt signaling pathway and recruits Etk to form a TNFR2-Etk-VEGFR2 complex that regulates cell growth and proliferation [27,28]. TNFR2 also appeared to have a T cell costimulatory function that is crucial for Treg and MDSC activation and growth in vitro [29,30]. Figure 2 illustrates the signaling pathways of TNF-TNFR2 in TME of CRC.

## 3. Tumor Necrosis Factor Receptor 2 (TNFR2): An Outlook in CRC

Upregulated production of TNFR2 on cancer cells could severely enhance the development of multiple myeloma, renal cell carcinoma, Hodgkin’s lymphoma, cutaneous non-Hodgkin’s lymphoma, ovarian cancer, and colon cancer [31]. For example, Zhao et al. have presented the effect of TNFR2 on proliferation, in such a way that the receptor successfully regulated the Ki-67 expression [32]. The growth of SW116 was highly proliferative in the presence of TNFR2, but the growth of HT29 was reduced when TNFR2 was silenced [32]. Furthermore, TNFR2 affects signaling pathways that are also important for the development and progression of tumors, such as P13K/AKT and MPAK/ERK. According to [32], TNFR2 participated in AKT signaling by increasing the activated phosphorylation of AKT, not ERK [32]. This indicates that TNFR2 can regulate the proliferation of CRC cells and promote the progression of CRC growth via P13K/AKT signaling pathway [32].

In addition, Hamilton et al. showed that TNFR2 overexpression increased the proliferation of two different colon cancer cell lines. Overexpression of TNFR2 in COLO205 cells promoted anchorage-independent growth. Although the effects of TNFR2 overexpression on proliferation and anchorage-independent growth seem to be minor, it is crucial to note that the colon cancer cell lines employed have high basal proliferation rates and are often resistant to growth increase in response to external stimuli [33]. The study further verified the induction of TNFR2 mRNA and protein by combined IL-6 and TNF, which offered direct evidence for the major function of STAT3 in TNFR2 induction. It also showed that TNF promoted IL-6 in SW480 cells, implying that TNF-induced IL-6 performs autocrine actions that contribute to the capacity of TNF and IL-6 to jointly drive TNFR2 expression [33]. The study established that TNFR2 was raised during the episodes of acute dextran sodium sulphate (DSS)-colitis, which was preceded by IL-6/STAT3 activation.

Mizoguchi et al. presented the first evidence for the upregulation of the TNFR2 during intestinal inflammation [34]. They also found that disrupting TNFR2 reduced the proliferation of intestinal epithelial cells (IEC) in a T-cell receptor (TCR) deficient colitis model [34]. Previous in vitro investigations had shown that both IL-6 and TNF were necessary to trigger TNFR2 in CRC cells, perhaps indicating a physiological microenvironment of several cytokines in inflammatory bowel disease (IBD) or IBD-associated CRC. As such, the involvement of the immunosuppressive immune cells (such as Treg cells and MDSCs) also contributes to poor CRC prognosis. The higher levels of FOXP3 Treg cells are associated with poor prognosis, indicating that FOXP3 Treg cells inhibit tumor immune surveillance and so promote tumor development and progression [35]. Furthermore, the presence of TNFR2 was seen on immunosuppressive cells such as Tregs, MDSCs, and also in the cancer cells. The expression of TNFR2 on these cells enhanced the suppressive functions and inhibited the antitumor action of effector cells, leading to tumor progression.

## 4. TNFR2 Expression by Treg Cells

Regulatory T cells (Tregs) are frequently associated with a poor prognosis in cancer patients and are abundantly infiltrated into tumor tissues; however, removing Tregs induces an anti-tumor immune response but at the same time can stimulate severe autoimmunity. Tregs play a major role in the establishment of immunosuppressive TME [36,37]. Indeed, Treg cells not only directly help tumor cells escape the fate of apoptosis, but they also help tumors survive by inhibiting the subset of CD8^+^ effector T cells [38]. The transcription factor FOXP3 plays an important role in the development and function of Treg cells. Treg cells that express high levels of FOXP3 (FOXP3 Treg cells) are significantly more suppressive than Treg cells that express low levels of FOXP3 [39]. Moreover, Treg cells express TNFR2 receptors, indicating that TNF-TNFR2 interaction is important for Treg cell biology, including activation, expansion, and phenotypes. A previous study reported that TNFR2 expression on Treg cells is very suppressive [30,40] and is associated with a poor prognosis in patients [41]. Another set of results revealed that an anti-TNFR2 antibody (TY101), in combination with HMGN1 (N1, a dendritic cell stimulating TLR4 agonist) and R848 (a synthetic TLR7/8 agonist) effectively depleted Tregs and boosted cytotoxic CD8 T cells in mouse colon tumors [42]. Yet another study found that a humanized antibody (AN3025) against human TNFR2 and cynomolgus TNFR2 effectively prevented the activation of human TNFR2 by competing with TNFα for binding to human TNFR2. This inhibition was further reflected by Tregs’ loss of function followed by the infiltration of CD4^+^ and CD8^+^ T cells to the site of the tumor [43]. Given the current scenario, anti-TNFR2 immunotherapy, either alone or in conjunction with anti-PD-1, significantly changed the TEM to eliminate tumor-specific Tregs and enhance Teff cells [44]. Furthermore, activated Tregs can produce a substantial quantity of sTNFR2, which enhances the immunosuppressive mechanism of Tregs [45]. As a result, elevated s-TNFR levels have been linked to disease progression in a number of cases in individuals with CRC compared to controls [46]. Meanwhile, TNFR2 can increase Treg cell activity and phenotypic stability [47]. Several studies have found that TNFR2^+^ Tregs increase primary tumor development and metastasis [47,48].

Furthermore, in the intracellular pathway of human Tregs, TNFR2 promotes IL-2-induced Treg proliferation and cell number expansion via the non-canonical NF-kB pathway [49]. Tregs also regulate immune suppression through a variety of mechanisms, including CTLA-4-mediated suppression of co-stimulatory (CD80/CD86) cells by dendritic cells, high affinity for IL-2 receptor compared to effector T cells, secretion of inhibitory cytokines, modulation of trytophan and adenosine, and induction of T effector apoptosis [50]. TNF increased the expression of CD25 and FOXP3, which promote the proliferation and suppressive functions of Treg cells [51]. Besides, other members of the TNFR family, such as GITR, OX40, and 4-1BB, are also expressed preferentially by Treg cells, and their expression is increased when the cells are in an active state [52]. To this extent, TNFR2, OX40, and GITR corresponded to the Treg cell signature in transcriptome analyses that compared Treg cells with conventional CD4^+^CD25^−^ T cells of lymphoid organs, and their expression correlated with reduced DNA methylation in Treg cells, indicating that their transcription is at least partly controlled at the epigenetic level [53]. These three molecules are expressed from the thymic Treg cell progenitor stage onwards in the Treg cell lineage, and their expression is required for Treg cell development [54]. Furthermore, the expression of TNFR2 distinguishes a subgroup of Treg cells with the greatest suppressive ability. The direct influence of TNF on TNFR2-expressing Treg cells has been investigated in various reports. TNF was able to enhance the proliferation, survival, and stability of Treg cells, as well as the expression of CD25, FOXP3, and activation markers and their suppressive activity [17,52]. For example, the effects of TNF on proliferation may be replicated using human Treg cells [55]. On the other hand, TNF decreased the suppressive function of human Treg cells in several investigations [56,57,58] and TNF can also make conventional T cells more resistant to Treg cell-mediated suppression [57]. Other than Tregs, TNFR2 signaling also regulates MDSC proliferation and suppressive action [59]. A previous study by Ba et al. showed that TNFR2 deficiency on MDSCs inhibits CXCR4 expression, whereas mTNF-expressed cancer cells attract these MDSCs into the TME [60]. Thus, TNFR2 is highly expressed on these immunomodulatory cells in the TME.

## 5. TNFR2 Expression on MDSCs

MDSCs are heterogeneous cell groups that act as effective repressors of the immune system. The cells may be located in the TME, peripheral blood, or bodily fluids, among other locations [61]. They also populate a multitude of malignancies, including the brain, breast, head and neck, lung, and many others. MDSCs go through several different developmental phases at different times. However, certain cancers produce substances that work to keep such cells in an immature condition. In MDSCs, this is accomplished by inhibiting the expression of a so-called “development pathway,” which prevents them from undergoing further development into antigen-presenting cells (APCs), such as dendritic cells (DCs) and macrophages in the TME [62]. In several studies, an increased MDSC count in peripheral circulation was associated with cancer resistance or refractoriness to immune-stimulating treatments, such as immunotherapies [63]. The prevalence of MDSCs within the tumor was linked to a higher risk of cancer recurrence in individuals with head and neck squamous cell carcinoma [64]. The effectiveness of adjuvant treatments is limited by the activity of these cells inside a tumor [64]. MDSCs migration and proliferation is one of the putative processes associated with immunotherapy failure in individuals with malignancies such as melanoma [65]. The measurement of these processes, together with that of Treg cells in the peripheral circulation, may be used as a prospective immunotherapy response biomarker [66].

TNF-TNFR2 interaction does not only appear to influence Treg cells, but it also affects MDSCs. In a chronic inflammation model produced by repeated administration of BCG, TNF-dependent immune suppression was linked to the formation of functional MDSCs [67]. In this model, splenic MDSCs of TNF-deficient mice generated less nitric oxide, had reduced arginase 1 function, and were less suppressive of T cell expansion. TNF also inhibited the development of myeloid splenocytes into mature macrophages and DCs. Zhao et al. reported that the TNF receptor controls MDSCs development in a cancer cell line, and although malignant rejection was found to be TNF-dependent and was correlated with the proliferation of MDSCs, TNFR2-deficient (TNFR2^−/−^) animals failed to promote MDSC formation and exhibited decreased cancer progression after malignant tumor implantation [68].

The same report found that TNF signaling promoted the recruitment of MDSCs in the peripheral blood. MDSC accumulation in the periphery was significantly reduced in TNFR2^−/−^ mice [68]. Furthermore, these findings revealed that TNFR2 signaling, rather than TNFR1 signaling, enhanced MDSCs survival by upregulating cellular FLICE-inhibitory protein and inhibiting caspase-8 activation. Interestingly, TNFR depletion hampered the production of MDSCs from bone marrow cells, although this could be restored by caspase inhibitor therapy [68]. These findings showed that TNFR2 signaling helps cancer cells escape the immune response by promoting MDSCs survival and recruitment. There is a growing body of evidence to suggest that MDSCs are involved in CRC development and progression [67]. Studies using mouse models showed that myeloid cells attracted by the CCL2-CCR2 signaling pathway might enhance CRC progression [69]. Moreover, CCL2 caused the recruitment of MDSCs and improved their immunosuppressive function during CRC carcinogenesis [70]. Notably, the suppressive activity of MDSCs in CRC is primarily linked to their capacity to block T cell proliferation while also stimulating Treg cell formation [71]. Another study demonstrated that TNFR2 production is essential for MDSCs recruitment during cancer development, and TNFR2 signaling is both reasonable and necessary for the MDSCs protection against apoptosis [68].

Additionally, the immunosuppressive role of the TME is one of the reasons for poor therapy response in CRC patients. Patients with advanced CRC develop resistance to chemotherapy, radiotherapy, immunotherapy, and targeted drug therapy, which results in increasing challenges in treating CRC. However, several prospective immunotherapies have been developed in a variety of cancer situations. Immune checkpoint-blocking antibodies are one of the promising approaches that have been demonstrated in CRC patients by inhibiting inhibitory immunological checkpoints and increasing immune responses against tumors.

## 6. Immune Checkpoint Inhibitors (ICI) and TNFR2

Immune checkpoint molecules with their cell surface receptors play critical roles in immune system modulation and maintain tolerance under normal conditions by providing inhibitory signals to T cells. Immunotherapy, particularly inhibitors targeting immune checkpoints, including cytotoxic T-lymphocyte antigen-4 (CTLA4), programmed cell death protein 1 (PD-1), and programmed cell death 1 ligand 1 (PD-L1), has provided promising new therapy to improve the overall survival of patients with CRC [50,69,72].

The inhibitory immunological checkpoint, cytotoxic T-lymphocyte-associated protein-4 (CTLA-4) is expressed on activated T cells and Treg cells. The infiltration Treg showed high expression of various immune checkpoint receptors that make it a more viable target for ICI [73]. Tregs activation by CTLA-4 is critical in avoiding autoimmunity [74]. CTLA-4 is also highly expressed on CD4^+^ Foxp3^+^ Treg and is necessary for Treg regulatory activity [75]. Additionally, blockage of CTLA-4 with mAbs (anti-CTLA-4) also provides a potential strategy for anticancer treatment by reducing Treg cell function that may enhance the host anti-tumor responses [76]. A study by Kamatham et al. showed that immune checkpoint blockade is a promising treatment option for those with mismatch repair deficient (dMMR)/microsatellite instability-high (MSI-H) mCRC [77]. Furthermore, the combination of anti-CTLA-4 (Tremelimumab) and anti-PD-L1 (Durvalumab) may improve overall survival (OS) in patients with advanced recurrent CRC and may be more effective than targeting anti-CTLA-4 alone in CRC [78].

The surface receptor, programmed cell death-1 (PD-1), plays a role in suppressing T cell anti-tumor activities and allowing tumor cells to evade the immune response. PD-1 suppresses T cells by interacting with its ligands (PD-L1) and PD-L2 (B7-DC). PD-L1 is expressed on many different cell types, including T cells, B cells, endothelium cells, and tumor cells [79,80]. Meanwhile, the expression of PD-L2 is limited and is only expressed on the surface of APCs and non-hematopoietic tissues. Moreover, blockage of PD-1 with anti-PD-1 may promote T cells anti-tumor activity; the two most common anti-PD-1s are Nivolumab and Pembrolizumab. A previous study showed that the use of Nivolumab in individuals with dMMR/MSI-H metastatic CRC found reliable responses in patients who had experienced prior treatments and the use of Nivolumab and other ICI was shown to have beneficial benefits in MSI-H mCRC treatment in phase I and II clinical studies [81,82]. Pembrolizumab (anti-PD-1) had a positive effect in CRC patients who expressed PD-L1, confirming the drug’s suitability in PD-L1-positive CRC patients [83]. In patients with previously treated MSI-H/dMMR mCRC, targeting PD-1 immune checkpoints with a combination of anti-PD-1 mAbs (Nivolumab with low-dose Ipilimumab) may provide an effective therapeutic approach [84].

Despite the effectiveness and advancements in cancer therapy achieved with ICI, side effects are one of the obstacles and limitations of ICI treatments. Immune-related adverse effects (irAEs) caused by ICIs are common in organs such as the skin, gastrointestinal tract, lung, kidney, and nervous system [85]. Managing irAEs is a crucial element of ICI therapy, and several treatments, such as corticosteroids, should be considered to prevent this side effect [86]. From this literature, ICI therapy has been shown to be effective, and also the efficacy of the combination of ICIs with another antibody is higher than that of a single antibody [44]. Apart from that, TNFR2 was also shown to have a potential as a therapeutic target, and this would enhance the anti-tumor effect to inhibit the development of cancers, including CRC.

**Figure 3:**
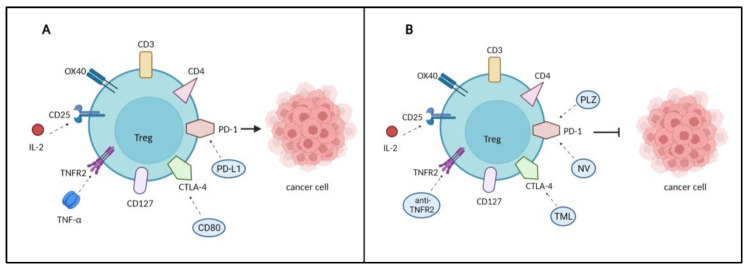
The expression of various markers on Treg. (**A**) The interaction of inhibitory immune checkpoints with their ligands impairs the antitumor activity of Treg and enhances the suppressor function of Treg and promotes cancer development. (**B**) Targeting inhibitory immune checkpoints with monoclonal antibodies (mAbs) as a potential targeted therapy in cancer treatment to inhibit cancer progression. PLZ: Pembrolizumab, NV: Nivolumab: TML: Tremelizumab.

To date, TNFR2-expressing Tregs or myeloid suppressor cells are the most effective immunosuppressive cells to inhibit the host immune response [87,88]. Tam et al. investigated the activity of murine and human anti-TNFR2 antibodies for cancer therapy. Their findings showed that the murine version had an antitumor impact on several cancer types and that human agonist TNFR2 antibodies had an equivalent activity that could be employed in patients [89]. A study by Case et al. showed that a combination of anti-TNFR2 with anti-PD-1 was most effective, and that anti-TNFR2 alone might be a potentially useful therapy for those who do not respond to or cannot tolerate anti-PD-1 or other checkpoint inhibitors [44]. The combination approach potentially increases a single agent’s treatment effectiveness and perhaps broadens the spectrum of indications when compared to monotherapies. Thus, TNFR2 has an attractive prospect since its expression on endothelial and immunosuppressive cells is crucial for both uncontrolled tumor angiogenesis and maintenance of an immunosuppressive TME [90]. For these reasons, TNFR2 inhibition in combination with an angiogenesis regulator may allow for both tumor vascularization regulation and a more robust antitumor response. Figure 3 visualizes the expression of surface markers on Treg and potential therapeutic targets in regulating tumor development.

## 7. Targeting TNFR2 in CRC Cancer

TNFR2 acts as both an oncogene and Treg- or MDSCs-cells inducer. Furthermore, its limited expression on other cell types renders this receptor a selective target for cancer treatment. In respect to CRC, TNFR2 is responsible for the promotion of proliferation and angiogenesis through several other mechanisms, such as by mediating progranulin [91] and STAT3 [33]. Currently, in a study with murine colon cancer models, the combination of anti-programmed death receptor-1 with new anti-TNFR2 led to complete tumor regression and elimination in more than 60% of the animals [44]. The mechanism behind this therapeutic potency is thought to involve the reduction of immunosuppressive Treg cells in the TME, thus increasing the ratio of the CD8^+^ effector cells to the Treg cells. The role of TNFR2 in CRC, however, is not only associated with its functional and preferential expression to Treg cells. Soluble TNFR2 (sTNFR2) reflects the upregulation of TNFR2 during inflammation, and the elevation of sTNFR2 was shown to increase the risk of CRC [92]. Additionally, higher sTNFR2 levels in patients with CRC are associated with higher mortality, although not CRC-specific mortality [93]. These roles of TNFR2 in the pathogenesis and progression of CRC render this receptor a potent target for CRC therapy. For cancers, including CRC, inhibition of TNFR2 or depletion of TNFR2-expressing cells can be achieved with the conventional antibody technology, and both in vitro and in vitro observations have been encouraging [44,57,89,94]. Although this approach could produce high-affinity anti-TNFR2, several limitations are of concern, including the unwanted FcγR-receptor side effects and the fact that it is only effective in the dominant manner of TNFR2 in the expansion of Treg cells [95]. TNFR2 is established to be preferentially expressed on Treg cells and important for their suppressive function; however, these characteristics are not exclusive to TNFR2 alone. Several other receptors, such as CD28 and CD25, as well as other TNFR receptors (GITR, OX40, 4-1BB), could also promote either the proliferation or the function of Treg cells, thus limiting the efficacy of targeting against any of these molecules. A better approach would be a system of targeting Treg cells as a whole by integrating all or most of their receptors. One such system is conceivably possible with the advent of nanotechnologies. Nanotechnologies have grown over the decades into a highly prospective tool in cancer therapy, where they are routinely used as drug and immune modulators and carriers that provide targeted delivery and prolonged accumulation in the organism [14].

Therefore, nanotechnologies may provide enhanced response rates and reduce the side effects that limit the full capacities of conventional drugs. Nanotechnology-based therapy can overcome the disadvantages of conventional therapy because of its small size, non-toxicity, biocompatibility, and distinct physicochemical properties [6]. Nanomaterials can be made comparable in size to biological macromolecules, making them ideal for cancer detection and treatment [6]. In addition, the advancement of nanotechnology-based medicinal approaches has resulted in significant improvements in the solubility of drugs as well as in their efficacy, bioavailability, and pharmacokinetics. As such, several nanomedicines have been approved by the FDA, such as liposomes [6] for cancer management, especially in the metastasis stage, and have been shown to fulfill their advantageous prospects, although there are still limited options existing for CRC [96].

## 8. Conclusions

Tissue immunosurveillance suppression is a primary mechanism that prevents the immune system from destroying malignant cells and reduces the effectiveness of current cancer treatments including radiation, chemotherapy, and immunotherapy. Furthermore, pro-tumorigenic immune cells in the TME promote the growth of cancer. Immunosuppressive cells such as Treg cells and MDSCs that interact with TNFR2 play a key role in cancer growth and progression because this interaction dampens the anti-tumorigenic immune cells by promoting the Treg cells and MDSCs suppressive activity.

For this reason, more anti-tumorigenic immune cells must be recruited to prevent cancer from developing. As a result of this recruitment, these cells become particularly susceptible to therapy. This effect will be important in cancer immunotherapy, as the immunological contexture of the TME shifts even more toward provoking a successful immune response. If conceived and implemented effectively, one such immunological therapeutic regimen for CRC will succeed in eradicating the cancer immune resistance, while simultaneously obtaining a long-term positive response to therapy.

## Data Availability

Not applicable.
